# Inhibition of *Borrelia Burgdorferi*-Induced TLR2-NFκB Canonical Signaling by Gallic Acid through Targeting the CD14+ Adaptor Protein and p65 Molecule

**DOI:** 10.3390/ijms231910987

**Published:** 2022-09-20

**Authors:** Anna Goc, Matthias Rath, Aleksandra Niedzwiecki

**Affiliations:** Department of Infectious Diseases, Dr. Rath Research Institute, San Jose, CA 95138, USA

**Keywords:** gallic acid, *Borrelia burgdorferi*, CD14+, NFκB, inflammation, Lyme disease

## Abstract

The cases of Lyme disease caused by *Borrelia burgdorferi* infection have been increasing throughout Northern America and Europe. This pathogen, if not treated in a timely manner with antibiotics, can cause persisting and debilitating health outcomes. In the search for novel agents against *B. burgdorferi*, we investigated a phenolic compound—gallic acid—for its anti-*Borrelia* and anti-inflammatory effects. Our results showed its biocidal effect starting from 100 μg/mL against active spirochetes, persisters/round-shaped bodies, and biofilm like aggregates of *B. burgdorferi sensu stricto*. Activation of macrophages by live *B. burgdorferi* also resulted in a robust NFκB-dependent proinflammatory responses seen in increased production of cytokines. Using human CD14+ macrophages *in vitro*, we showed that CD14+ adaptor and phosphorylated p65 molecule are impeded at nonbiocidal and noncytotoxic concentrations of gallic acid, resulting in the inhibition of both expression and secretion of cytokines IL1β, IL6, and TNFα. Our findings demonstrate efficacy of gallic acid against *B. burgdorferi* and provide potential mechanistic insight into its TLR2/CD14+-NFκB mediated mode of action. Further studies on the potential of gallic acid as a safe and effective compound against *Borrelia-*caused infection are warranted.

## 1. Introduction

*Borrelia burgdorferi sensu lato* is the causative pathogen of Lyme disease (LD), the most common vector-borne disease in Europe and North America [1,2]. It is an invasive, host-dependent and semi-aerophilic bacterium with three predominant morphological forms [1,2,3]. The vegetative (active) form is the motile spirochetes, which under conditional stress can quickly transform into persistent forms of knob-/round-shaped bodies and/or biofilm like aggregates. The latent forms of *B. burgdorferi sensu lato* are acknowledged to be responsible for a persisting type of LD in humans, although this aspect remains controversial [4,5,6,7]. LD is a multisystemic zoonosis with inflammatory responses at its basis, affecting multiple organs. The pathogenesis in early stages of LD is associated largely with the presence of viable bacteria at the site of inflammation, whereas in the later stages of LD, inflammatory and autoimmune features seem to prevail.

*B. burgdorferi* is an extracellular pathogen with pathogen-associated molecular patterns (PAMPs), such as lipoproteins and nucleic acids, serving as the toll-like receptor 2 (TLR2) ligands, and inducing proinflammatory cytokine production [8,9]. This signaling cascade engages the adaptor protein CD14+ as an acknowledged element in mediating proinflammatory responses and immunomodulatory–immunosuppressive features [10,11]. Involvement of the canonical factor kappa B complex (NFκB) signaling pathway seems to be plausible as well [10,12,13]. NFκB is a family of Rel transcription factors (i.e., NFκB1, NFκB2, RelA/p65, RelB, and c-Rel) encoded by five genes: NFκB1, NFκB2, RELA, RELB and c-REL [13]. Proteins NFκB1 and NFκB2 are translated as large precursor proteins known as p105 and p100 that undergo proteolysis to become p50 and p52 respectively, whereas RelA, RelB, and c-REL proteins contain a transactivation domain (TAD) in their C-terminal region. Thus, they can induce DNA transcription, while the p50 and p52 homodimers are able to repress it by merely subjugating the DNA binding sites and blocking its transcription [13,14]. In the canonical pathway, NFκB comprises subunits p65 and p50 and exists in two forms: active (phosphorylated), acting in the nucleus, and inactive (dephosphorylated), present in the cytoplasm [15,16,17], where it undergoes the activation by cell stressors [18,19,20]. This activation occurs through several intermittent steps that lead to an activation through phosphorylation of the IκB kinase (IKK) complex, which subsequently results in its ubiquitination and degradation. Once degraded, the remaining NFκB dimer (e.g., p65/p50 subunits) translocates to the nucleus, where it activates transcription of numerous target genes.

Gallic acid (3,4,5-trihydroxybenzoic acid) is a low molecular triphenolic compound existing in monomeric as well as in polymeric form. Its mode of antimicrobial action, although not fully established, may be attributed to deactivation of a plethora of microbial proteins, including adhesins, enzymes and cell envelope transport proteins [21,22,23]. Gallic acid was found to show synergism with several existing chemotherapeutic drugs, as well as to serve as an anchor for the design or development of new pharmacological agents with diverse therapeutic features [24].

In this study, we tested the anti-*Borrelia* and anti-inflammatory effects of gallic acid. The anti-*Borrelia* effect was examined by utilizing a high-throughput screening method allowing for assessing viability of the logarithmic phase (rich in typical motile spirochetes) and stationary phase (rich in knob-/round-shaped persisters). The anti-inflammatory effect of gallic acid was studied against *B. burgdorferi*-induced inflammation in CD14+ macrophages, focusing on proinflammatory cytokine production and secretion, and the mode of action. To address this particular aspect, we focused on the toll-like receptor 2 (TLR2)–NFκB canonical signaling pathway, evaluating the potential of gallic acid to alter the levels of its key mediators. Here, we report that gallic acid significantly decreases the levels of cytokines relevant in LD, such as IL1β, IL6 and TNFα induced by live *B. burgdorferi*, through downregulation of the CD14+ adaptor and TLR2, and further downstream decreasing level of phosphorylated p65 molecule.

## 2. Results

### 2.1. Anti-Borrelia Efficacy of Gallic Acid

**Figure 1:**
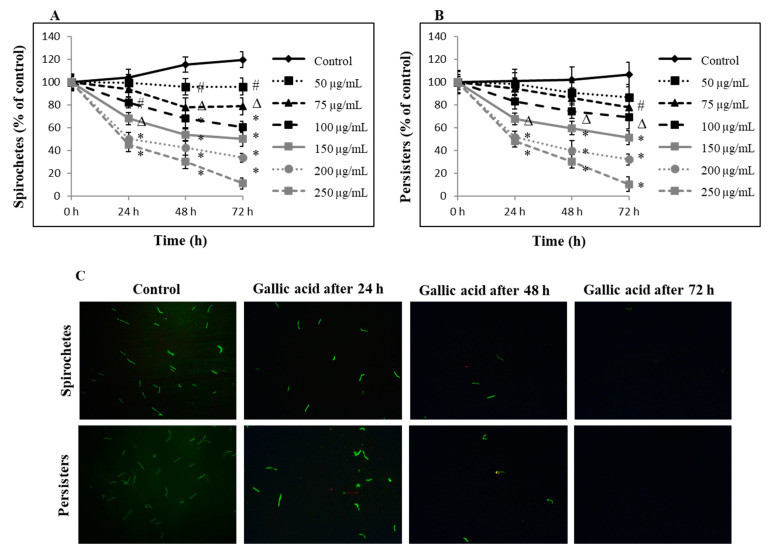
Kinetic evaluation of biocidal effect of gallic acid. Dose-dependent efficacy of gallic acid against spirochetes and persisters of *B. burgdorferi* B31 was monitored up to 72 h and determined by SYBER Green I/IP assay using spectrofluorescent measurement (**A**,**B**). Representative images of live/dead images of spirochetes and persisters of *B. burgdorferi* B31 stained with SYBER Green I/IP day (**C**). Images taken at 200× magnification; # *p* ≤ 0.05, ∆ *p* ≤ 0.01, * *p* ≤ 0.001; control—DMSO only (0.125%).

**Figure 2:**
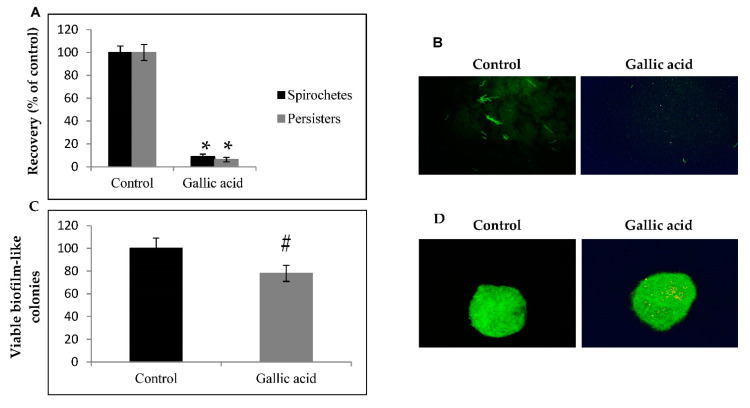
Effect of gallic acid on latent forms of *B. burgdorferi*. Estimation of repopulated spirochete form of Borrelia burgdorferi (**A**). *B. burgdorferi* B31 was treated with gallic acid (250 µg/mL) for 72 h and transferred to fresh tubes containing medium only. After 14 days of subculturing, the presence of typical motile spirochetes was determined by SYBR Green I/PI assay using spectrofluorescent measurement. Representative live/dead images of *B. burgdorferi* B31 exposed to 250 µg/mL of gallic acid after 14 days of subculturing (**B**). Images taken at 200× magnification; susceptibility of *B. burgdorferi* B31 biofilm to gallic acid (**C**). Biofilm like structures were exposed to 250 µg/mL of gallic acid for 72 h. Viability was assessed by SYBER Green I/IP staining method. Representative live/dead images of *B. burgdorferi* B31 biofilm exposed to 250 µg/mL of gallic acid for 72 h. Images taken at 400× magnification (**D**); # *p* ≤ 0.05, * *p* ≤ 0.001; control—DMSO only (0.125%).

Biocidal efficacy of gallic acid against typical motile spirochetes and knob-shaped persisters of *B. burgdorferi* B31 strain are presented in Figure 1. The results showed a dose-dependent and time-dependent biocidal effect of gallic acid against motile spirochetes ranging from 5.0–60% after 24 h, 10–65% after 48 h, and 12–98% after 72 h µg/mL (Figure 1A,C). A time-dependent killing effect on the latent knob-shaped persisters was also observed that oscillated between 2.0–50% after 24 h, 9.0–61% after 48 h and 10–97% after 72 h of exposure (Figure 1B,C). These values corresponded to each other for both tested morphological forms of *B. burgdorferi.* Further study validated the biocidal activity of gallic acid by conducting an experiment, in which motile spirochetes treated with gallic acid at the 250 µg/mL that had earlier shown to be 95–99% biocidal, were allowed to recover in the medium without gallic acid addition. Performed examination revealed the presence of about 11% of recovered motile spirochetes (Figure 2A,B). The same concentration showed 23% biocidal activity in biofilm like colonies (Figure 2C,D).

The triple combination of antibiotics (doxycycline + daptomycin + cefoperazone) at the concentration of 30 µg/mL (10 µg/mL, each) was used in our previous study as well by other research groups and showed about 90% of biocidal effect against spirochetes and persisters after 72 h incubation, but was ineffective against biofilm like aggregates grown on collagen surface, and treatment with this triple antibiotic combination resulted in ~11% regrowth of viable cells [25,26,27,28,29,30,31,32,33].

### 2.2. Anti-Inflammatory Effect of Gallic Acid against Borrelia burgdorferi-Induced In Vitro Infection

**Figure 3:**
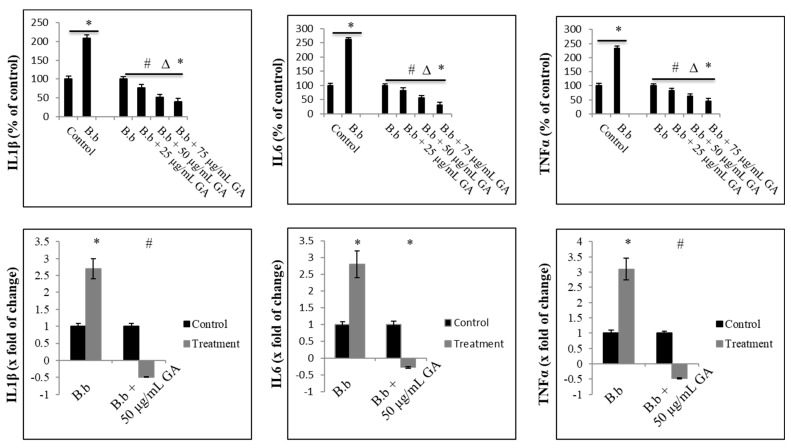
Proinflammatory cytokine secretion and expression status. Human CD14+ monocytes were treated with gallic acid (GA) upon *B. burgdorferi* 31 stimulation. Levels of secreted proinflammatory cytokines were assessed using ELISA after 12 h post-treatment (**upper panel**) and with RT-qPCR after 24 h post-treatment (**lower panel**). Control—cells not stimulated with *B. burgdorferi*; B.b —control cells stimulated with *B. burgdorferi* at cells:bacteria 1:5 ratio; # *p* ≤ 0.05, ∆ *p* ≤ 0.01, * *p* ≤ 0.001; control—DMSO only (0.035%).

The anti-inflammatory effect of gallic acid against CD14+ macrophages was evaluated by analyzing production and secretion of cytokines significant in the initial phase of LD by leukocyte exposure to live *B. burgdorferi*. The analysis indicated that activation with *B. burgdorferi* at MOI 1:5 resulted in a significant IL1β, IL6, and TNFα secretion, inhibited by gallic acid. Gallic acid significantly reduced the levels of cytokine secretion induced by live *B. burgdorferi* in CD14+ macrophages in dose-dependent fashion (Figure 3 upper panel).

Upon treatment with 25–75 µg/mL of gallic acid, the decrease in the secretion of IL1β ranged between 22 and 49%, whereas the decrease in secretion of IL6 and TNFα 10–45% was in a narrower range and oscillated between 17 and 44%. In addition, gallic acid inhibited expression of these cytokines at RNA level at about 0.48 magnitude of change for IL1β, 0.29 magnitude of change for IL6, and 0.48 magnitude of change for TNFα (Figure 3 lower panel).

**Figure 4:**
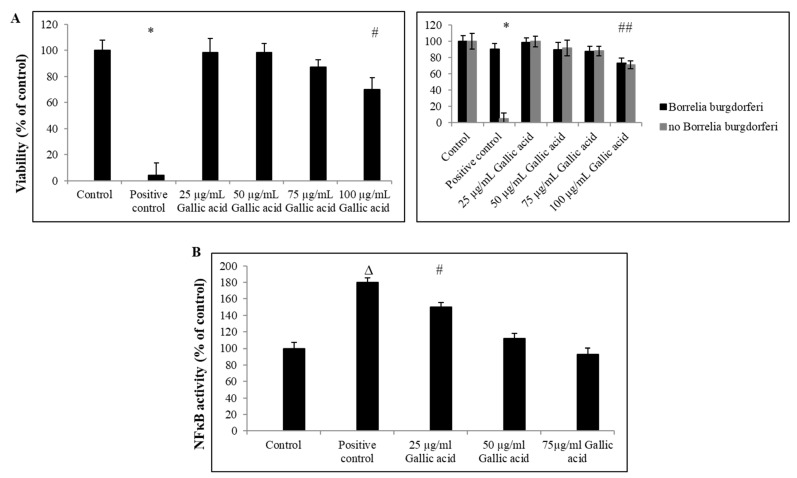
Effect of gallic acid on NFκB activity. Viability of CD14+ monocytes (**A**, **left panel**) and NFκB reporter cells (**A**, **right panel**) upon treatment with *B. burgdorferi*. 1—control cells not stimulated with *B. burgdorferi*; Positive control—control cells stimulated with *B. burgdorferi* at cells:bacteria 1:5 ratio or 100% dead cells; control—DMSO only (0.05%). Activity of NFκB (**B**). NFκB reporter cells were treated with gallic acid upon PMA stimulation. Activity was assessed using NFκB activity ELISA kit after 24 h post-treatment. 1—control cells not stimulated with PMA; positive control—control cells stimulated with PMA at 3 nM concentration; # *p* ≤ 0.05, ∆ *p* ≤ 0.01, * *p* ≤ 0.001; control—DMSO only (0.035%).

We observed that gallic acid did not reveal cytotoxicity towards CD14+ cells up to 75 μg/mL (Figure 4A). In addition, as presented in Figure 4B, the activity of NFκB reported cells decreased upon gallic acid treatment at 25–75 µg/mL concentrations.

**Figure 5:**
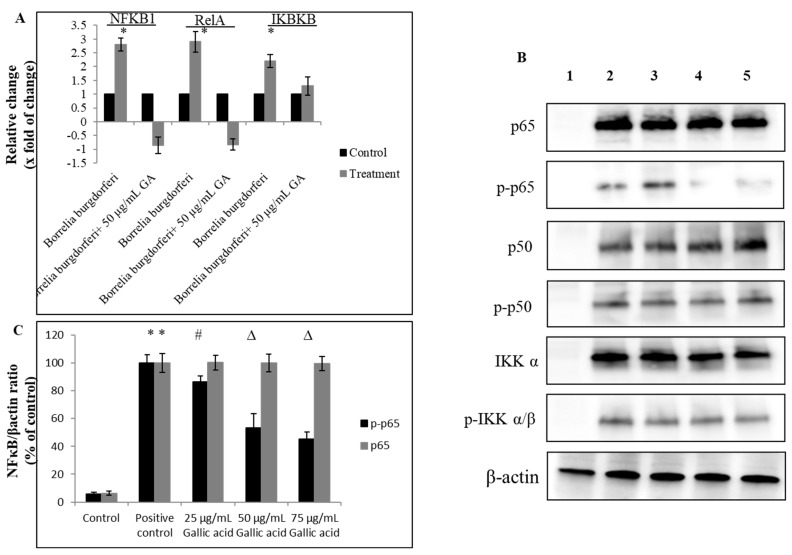
Effect of gallic acid on NFκB canonical signaling pathway. Human CD14+ monocytes were treated with gallic acid (GA) upon *B. burgdorferi* 31 stimulation. Levels of proteins were assessed by RT-qPCR (**A**) and Western blot (**B**) after 12 h post-treatment. Densitometry of Western blot bands (**C**). 1—control cells not stimulated with *B. burgdorferi*; 2—control cells stimulated with *B. burgdorferi* at cells:bacteria 1:5 ratio; 3—cells stimulated with *B. burgdorferi* at cells:bacteria 1:5 and treated with gallic acid at 25 µg/mL; 4—cells stimulated with *B. burgdorferi* at cells:bacteria 1:5 and treated with gallic acid at 50 µg/mL; 5—cells stimulated with *B. burgdorferi* at cells:bacteria 1:5 and treated with gallic acid at 75 µg/mL; # *p* ≤ 0.05, ∆ *p* ≤ 0.01, * *p* ≤ 0.001; control—DMSO only (0.035%).

In the CD14+ cells incubated with live *B. burgdorferi*, gallic acid applied at noncytotoxic concentrations (i.e., 75 μg/mL) reduced level of phosphorylated p65 protein but not total p65 molecule in the range of 19–59%, nor did it affect other components of the NFκB canonical pathway such as p50 and IKKα/β and their expression levels, including p65 protein (Figure 5 and Figure S1). Despite being overall activated by *B. burgdorferi*, the key components of the TLR2/CD14+ pathway, such as TLR2 and its adaptor molecule CD14+, were neither significantly upregulated or downregulated at both mRNA and protein levels.

**Figure 6:**
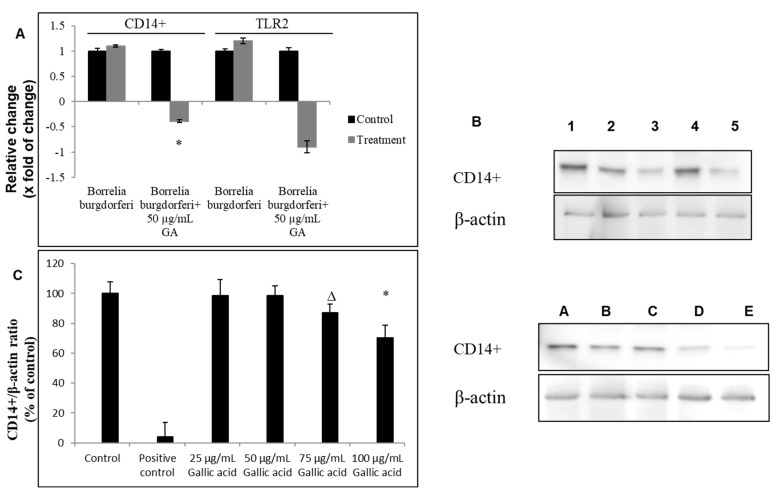
Effect of gallic acid on toll-like receptor 2. Human CD14^+^ monocytes were treated with gallic acid (GA) upon *B. burgdorferi* 31 stimulation. Levels of proteins were assessed by RT-qPCR (**A**) and Western blot (**B**) after 12 h post-treatment. Densitometry of Western blot bands (**C**). 1—control cells not stimulated with *B. burgdorferi*; 2—control cells stimulated with *B. burgdorferi* at cells:bacteria 1:5 ratio; 3—cells stimulated with *B. burgdorferi* at cells:bacteria 1:5 and treated with gallic acid at 50 µg/mL; 4—cells stimulated with *B. burgdorferi* at cells:bacteria 1:10; 5—cells stimulated with *B. burgdorferi* at cells:bacteria 1:10 and treated with gallic acid at 50 µg/mL; A—control cells not stimulated with *B. burgdorferi*; B—control cells stimulated with *B. burgdorferi* at cells:bacteria 1:5 ratio; C—cells stimulated with *B. burgdorferi* at cells:bacteria 1:5 and treated with gallic acid at 25 µg/mL; D—cells stimulated with *B. burgdorferi* at cells:bacteria 1:5 and treated with gallic acid at 50 µg/mL; E—cells stimulated with *B. burgdorferi* at cells:bacteria 1:5 and treated with gallic acid at 75 µg/mL; ∆ *p* ≤ 0.01, * *p* ≤ 0.001; control—DMSO only (0.035%).

However, gallic acid inhibited expression of CD14+ adaptor molecule at mRNA about 0.21 magnitude of change. Inhibition of CD14+ at protein levels was observed about 22–44% whether cell:bacteria ratio of 1:5 or 1:10 was applied (Figure 6 and Figure S2).

## 3. Discussion

LD, and especially its chronic manifestation, still poses a large therapeutic challenge. Currently, the first choice of LD treatment is based on antibiotics applied either as monotherapy or as combined therapy in conjunction with other antibiotics or adjuvant treatments with steroids, which result in both favorable and detrimental outcomes [34,35,36,37,38,39,40]. In search for other, nontoxic approaches, we evaluated *in vitro* efficacy of gallic acid against all acknowledged morphological forms of *B. burgdorferi* and its potential mechanisms of action in curbing *Borrelia*-induced inflammation. Our approach was dictated by the premise of successful eradication of *B. burgdorferi*, as well as decrease of inflammatory processes, which accompany this infection, as it negatively affects multiple organ systems, including the nervous system, cardiovascular system, joints, and muscles.

Polyphenols and their metabolites have been a source of natural agents with therapeutic activity and apparent health benefits in humans and animals for a long time. However, only a limited number of these natural compounds, with proven anti-*Borrelia* efficacy, have been identified [28,29,30]. We have previously shown that polyphenols such as baicalein and luteolin, as well as fatty acids such as monolaurin and 10-HAD, used at concentrations 200–500 µg/mL could reduce biofilm like colonies formed by *B. burgdorferi sensu stricto* by 30–60% [28]. However, only baicalein and monolaurin applied at these concentrations were effective in reducing biofilm like colonies formed by *B. garinii* by approximately 40–60%. Our subsequent studies identified various other polyphenols as having bacteriostatic and/or bactericidal effects against spirochetes [30]. However, their biocidal efficacy against rounded forms could reach only 30–50%, and they did not demonstrate any significant antibiofilm effects. However, based on these studies we concluded that phenyl groups in these compounds are important for their comprehensive antibacterial activity. Gallic acid is classified as phenolic acid, thus in the current study we focused on this compound, which in addition to overall safety as compared to synthetic drugs, can be metabolized easily and demonstrated anti-inflammatory effects [21]. Thus, in addition to examining its direct anti-*Borrelia* potential, we also tested its efficacy in curbing inflammatory response induced by live *B. burgdorferi sensu stricto.*

We observed a significant, dose- and time-dependent antimicrobial effect of gallic acid against both active and persistent forms of *B. burgdorferi,* including 23% biocidal effect against biofilm like colonies. This particular effect of gallic acid is of importance as the perseverance of viable organisms in latent forms might explain treatment failure and persistent symptoms following antibiotic therapy of LD, which otherwise is usually effective against spirochetes [26]. Reported studies suggest that bacteria within the biofilm like colonies could become 1000 times more resistant to antibiotics than other forms [26]. These adherent polysaccharide-based matrices are also responsible for other chronic infections, including periodontitis, endocarditis, gastrointestinal infection, and chronic lung infection, in which some beneficial anti-inflammatory effects of gallic acid were implicated [21].

Second aspect of this study included gallic acid anti-inflammatory effects, which to our knowledge were not studied in respect to LD. Inflammation accompanying *Borrelia-*triggered infection has a causal role in the pathogenesis of Lyme arthritis, as well as carditis, neurologic deficits called Lyme neuroborreliosis, and cognitive effects [10]. Its central feature is an excessive, dysregulated proinflammatory immune response during the infection phase that persists into the postinfectious period. CD14+ macrophage-mediated inflammatory responses are typically triggered by pathogen-associated molecular patterns (PAMPs) recognized by toll-like receptors (TLRs) [41,42,43,44,45]. Tanaka et al. showed that a natural plant extract rich with gallic acid, ellagic acid and gallate esters was protective against inflammation and oxidative stress by suppressing MAPK/NF-*κ*B pathway and by activating Akt/AMPK/Nrf2 pathway [23]. However, since plant extracts contain numerous active ingredients, the observed effects may be also associated with other compounds. We observed a dose- and time-dependent antibacterial effect of gallic acid against both active and persistent forms of *B. burgdorferi*. While higher concentrations of gallic acid (i.e., 100–250 μg/mL) resulted in the gradually elevated levels of dead forms, its lower concentrations (i.e., up to 75 μg/mL) facilitated a significant decrease in production and secretion of cytokines and their signaling mediators.

Our study showed inhibitory effect of gallic acid on the TLR2/CD14+-NFκB signaling axis triggered by live *B. burgdorferi*, which resulted in the inhibition of proinflammatory cytokines IL1β, IL6, and TNFα as relevant proinflammatory mediators in a clinical manifestation of LD [10]. It is important to note that NFκB signaling cascade triggers a variety of inflammatory factors and regulatory proteins depending on whether CD14+ is involved, thereby mediating a plethora of inflammatory processes [12,41,42,43,44,45,46]. Using in vitro human CD14+ macrophages, we noticed that gallic acid inhibits expression of the CD14+ adaptor protein. We also identified gallic acid as a compound constricting activation of NFκB signaling and CD14+ macrophages, which induce IL1β, IL6, and TNFα production. These findings allowed us to identify gallic acid as a compound that by acting through CD14+-dependent and CD14+-independent signaling, might lead to enhanced spirochetes clearance. Culture supernatants of CD14+ macrophages that were incubated with live *B. burgdorferi* at an MOI of 1:5 for 12 h showed significantly elevated levels of proinflammatory cytokines, which were among others, inhibited in the presence of gallic acid without increased levels of apoptosis. A specific role for gallic acid in *B. burgdorferi*-induced apoptosis and its potential to modulate this effect is worth exploration. It is possible, however, that these signaling pathways intertwine, mediating both inflammation and apoptosis and even phagocytic clearance of *B. burgdorferi*. Our data pointing out that gallic acid used up to 50 µg/mL for 22–24 h did not negatively influence viability of CD14+ macrophages and NFκB reporter cells. Tanaka et al. also did not observed cytotoxicity of gallic acid up to 50 µg/mL on RAW 264 macrophages [23]. Interestingly, Zhao et al. reported that gallic acid has selective dose-dependent cytotoxicity for cervical cancer cells, without injuring normal cells [47]. Thus, an implication of gallic acid as an inhibitory compound is plausible, and whether equivalent concentrations can be attained with clinical use of these agents in vivo remains to be determined.

In summary, our findings demonstrate a direct efficacy of gallic acid against *B. burgdorferi* and triggered by it, inhibitory effect on the CD14+/TLR2-NFκB-controlled proinflammatory response relevant in LD. We are also presenting a potential mechanistic insight into this process by showing the inhibitory effects of gallic acid on CD14+/TLR2-NFκB signaling that drives spirochete’s induced inflammation. Our results also confirm that the CD14+ macrophages have a particularly important role in clearance of *B. burgdorferi* and the immunologic response to spirochete infection. The documented anti-*Borrelia* efficacy of gallic acid provides scientific basis for its further evaluations including clinical applications as the spread of LD continues to increase on a global scale.

## 4. Materials and Methods

### 4.1. Test Compound

Gallic acid with a purity of 98%, according to the manufacturer was obtained from Sigma (St. Louis, MO, USA), and its stock solution (100 mg/mL) was prepared in 50% DMSO sterilized by 0.22 µm syringe filtration.

### 4.2. Borrelia burgdorferi Culture

Low passage isolate of the B31 strain of *B. burgdorferi* was obtained from the American Type Culture Collection (Manassas, VA, USA). The B31 strain is an isolate from *Ixodes dammini* and prepared for testing according to previous reports [7,25,26,27]. Briefly, the cryostocks of both species were cultured under commonly used conditions, i.e., Barbour–Stoner–Kelly H (BSK-H) medium, supplemented with 6% rabbit serum (Sigma, St. Louis, MO, USA), without antibiotics, at 33 °C with 5% CO_2_, in 15 mL polypropylene sterile test tubes. Homogeneous logarithmic culture (having only typical motile spirochetes/active form) of *B. burgdorferi* was obtained by maintaining inoculum in a shaking incubator at 33 °C and 250 rpm for 2–3 days. Stationary culture (enriched in knob-/round-shaped cells/persistent forms) of *B. burgdorferi* was generated by maintaining inoculum in an incubator at 33 °C for 7–8 days. Biofilm like aggregates of *B. burgdorferi* were prepared by incubation of inoculums in four-well chambers (BD Biosciences, Sparks, MD, USA) coated with collagen type I from rat tail for approximately 7 days.

### 4.3. Evaluation of the Biocidal Efficacy of Gallic Acid against Borrelia burgdorferi

Testing was assessed as previously reported [28,29,30,31]. Briefly, 96-well plates with 0.1 mL BSK-H medium were inoculated with 2 × 10^5^–1 × 10^6^ cells/mL (logarithmic phase, i.e., 2–3 days culture, or stationary phase, i.e., 7–8 days) and supplemented with the test compound. Control wells were treated with DMSO (0.125%). Next, the plates were incubated at 33 °C with 5% CO_2_ for 72 h. Assessment was done using high-throughput spectrofluorometric screening with SYBER Green I/IP staining (Nikon, Eclipse E600, Tokyo, Japan), respectively. The excitation wavelength was set at 485 nm and the absorbance wavelength at 535 nm. The biocidal effect of the gallic acid against biofilm like aggregates was evaluated in four-well chambers coated with collagen type I from rat tail as described [6,29]. Briefly, 1 × 10^7^ cells/mL was inoculated into each sterile chamber filled with 1 mL BSK-H medium and incubated for one week at 33 °C with 5% CO_2_, followed by 72 h of incubation with the test compound. Control wells contained DMSO (0.125%). Earlier studies in our laboratory have documented a lack of antifungal carryover using this procedure [28]. For quantitative assessment, all wells were fixed with 500 µL of cold formalin acetic acid mixture for 20 min, followed by staining with 200 µL of SYBER Green I/IP staining mixture for 15 min in the dark [29,31]. Pictures were immediately taken from untreated and treated mounted slides using a fluorescence microscope (Nikon, Eclipse E600). Results are expressed as a percentage of gallic acid-free control (mean +/− SD, *n* = 4).

### 4.4. Analysis of Cytokine Secretion

Proinflammatory cytokine release was assessed using the Human Cytokines ELISA array assay kit (Qiagen, Germantown, MD, USA) according to the manufacturer’s protocol. Briefly, 100 µL of supernatant of CD14+ macrophages (ATCC, Manassas, VA, USA) was incubated for 2 h in 96-well plates at RT with gentle shaking. After the incubation period, all wells were washed three times with 1 × Assay Wash Buffer and again incubated with 100 µL of biotin-labeled antibody mixture for 1 h at RT with gentle shaking. Next, the washing step was repeated, and all wells were again incubated with streptavidin-HRP conjugated for 45 min at RT with gentle shaking followed by the washing step. Signal was developed with TMB substrate followed by adding stop solution. Optical density was measured within 30 min at 450 nm. Results are expressed as a percentage of gallic-acid-free control (mean +/− SD, *n* = 4).

### 4.5. NFκB Activity Assay

NFκB activity was performed according to Indigo Biosciences assay kit (State College, PA, USA). Briefly, a 21 mL suspension of NFκB reporter cells was dispensed into a 96-well plate (200 µL per well) and preincubated in a cell culture incubator (37 °C, 5% CO_2_) for 4–6 h. The medium was then removed, and cells were rinsed with cell screening medium (CSM) and replaced with stock solution of gallic acid diluted with CSM to the desired final assay concentrations. Phorbol 12-myristate 13-acetate (PMA) at 3 nM, as well as a ‘no treatment’ control, were included. The plates were transferred into a culture incubator for 22–24 h. Subsequently, the treatment medium was discarded and 100 µL of luciferase detection reagent (LDR) was added to each well of the assay plate. After 5 min, chemiluminescence was measured. Results are expressed as a percentage of gallic-acid-free control (mean +/− SD, *n* = 4).

### 4.6. RT-qPCR

Genome copies of target genes in CD14+ cells (ATCC, Manassas, VA, USA) were quantified by extracting RNA by use of a RNeasy Plus Kit (Qiagen, Germantown, MD, USA), according to the manufacturer’s protocol. Extracted RNA was then taken for cDNA synthesis using the QuantiTect Reverse Transcription kit following the manufacture’s protocol (Qiagen, Valencia, CA, USA). Two-step RT-qPCR using QuantiTect SYBR Green PCR kit (Qiagen, Valencia, CA, USA) was performed for each sample in triplicates with PrimeTime qPCR Primer Assays (Qiagen, Valencia, CA, USA), and BioRad CFX instrument (Hercules, CA, USA), with cycling parameters as: 5 min at 95 °C; and 40 cycles at 95 °C for 10 s and 60 °C for 30 s. Genomic copies were normalized to the relative expression of the human β-actin gene using PrimeTime qPCR Primer Assay (Qiagen, Valencia, CA, USA).

### 4.7. Western Blot

CD14+ cells were lysed using lysis buffer [50 mM Tris-HCl (pH = 7.4), 1.0%] Triton X-100, 150 mM NaCl, 1.0 mM EDTA, 2.0 mM Na_3_VO_4_, and 1 X complete protease inhibitors (Roche Applied Science, Indianapolis, IN, USA)]. The protein concentration was measured by the Dc protein assay (Bio-Rad, Hercules, CA, USA). The protein samples (50 µg/well) were separated on 8–16% gradient SDS-PAGE gels (i.e., Tris-based electrophoresis using the standard Laemmle’s method) and transferred to a PVDF membrane. Specific proteins were detected either with commercially available anti-CD14+ antibody at 1:1000 dilution, anti-p65 and anti-p-p65 antibodies at 1:1000 dilution, anti-p50 and anti-p-p50 antibodies at 1:1000 dilution, anti-IKK α and anti-p-IKK α/β antibodies at 1:1000 dilution, and anti-β-actin antibody at 1:2500 dilution serving as a loading control. WB images were acquired using the Azure cSeries system and autoexposure settings (Azure Biosystems, Dublin, CA, USA). Original Western blot images are located in Supplementary Information file.

### 4.8. Viability Assay

Cellular viability was assessed using MTT assay according to the manufacturer’s protocol. Briefly, cells were plated in 96-well plates at 1 × 10^5^ cells per well in RPMI containing 10% FBS. After 24 h, the medium was replaced with the same medium supplemented with the gallic acid (0–100 µg/mL). After 24 h of treatment, cell viability was measured at 570 nm, using an ELISA reader (Molecular Device, Spectra Max 340, San Jose, CA, USA). Results are expressed as a percentage of gallic-acid-free control (mean +/− SD, *n* = 8).

### 4.9. Statistical Analysis

Data for all experiments, unless indicated otherwise, are presented as an average value and standard deviation from at least three independent experiments, each at least in three replicates. Comparison between different samples was done by a two-tailed *t*-test using the SPSS data analysis software. Differences between samples were considered significant at *p* values lower than 0.05.

## Data Availability

All data are included in this publication.

## Supplementary Materials

The following supporting information can be downloaded at: https://www.mdpi.com/article/10.3390/ijms231910987/s1.
