# Supplementary material for: Inhibition of Borrelia Burgdorferi-Induced TLR2-NFκB Canonical Signaling by Gallic Acid through Targeting the CD14+ Adaptor Protein and p65 Molecule
Source: Int J Mol Sci. 2022 Sep 20;23(19):10987. doi: 10.3390/ijms231910987 (PMC9570260; doi:10.3390/ijms231910987)
Supplement: Supplementary file 1 [file ijms-23-10987-s001.zip › ijms-1915102-supplementary.pdf]

Supplementary Information

Borrelia burgdorferi-induced TLR2-NFκB canonical signaling is inhibited by gallic acid through targeting the CD14+ adaptor protein and p65 molecule

Anna Goc[1*], Aleksandra Niedzwiecki[1*], Matthias Rath[1]
[1]Dr. Rath Research Institute, 5941 Optical Ct., San Jose, CA 95138, USA

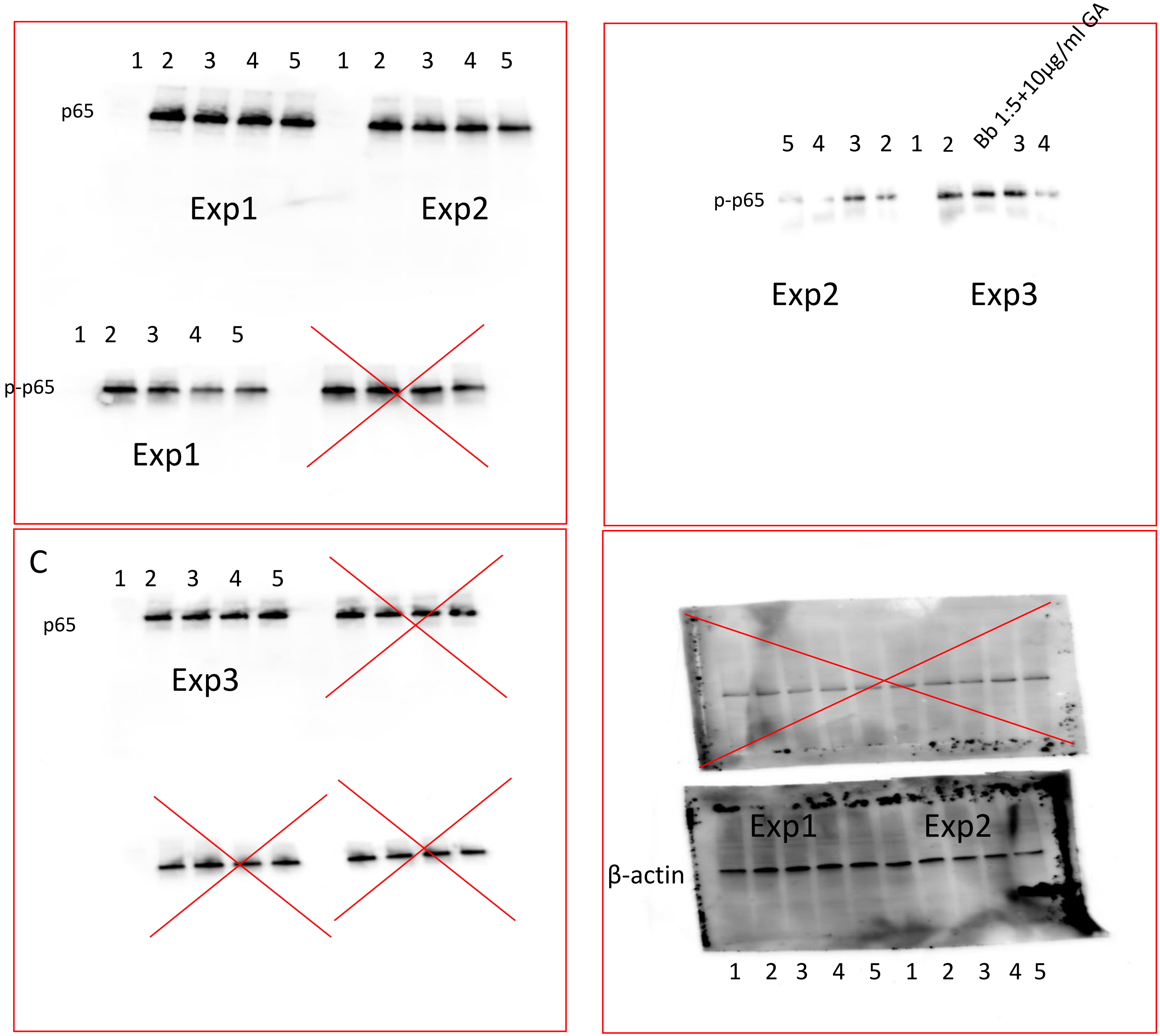

Figure S1a. Effect of gallic acid on NFκB canonical signaling pathway. Human CD14+ monocytes were treated with gallic acid upon *Borrelia burgdorferi* 31 stimulation. Levels of proteins were assessed Western blot after 12 h post-treatment. 1 - control cells not stimulated with *Borrelia burgdorferi*; 2 - control cells stimulated with *Borrelia burgdorferi* at cells:bacteria 1:5 ratio; 3 - cells stimulated with *Borrelia burgdorferi* at cells:bacteria 1:5 and treated with gallic acid at 25 µg/ml; 4 - cells stimulated with *Borrelia burgdorferi* at cells:bacteria 1:5 and treated with gallic acid at 50 µg/ml; 5 - cells stimulated with *Borrelia burgdorferi* at cells:bacteria 1:5 and treated with gallic acid at 75 µg/ml.

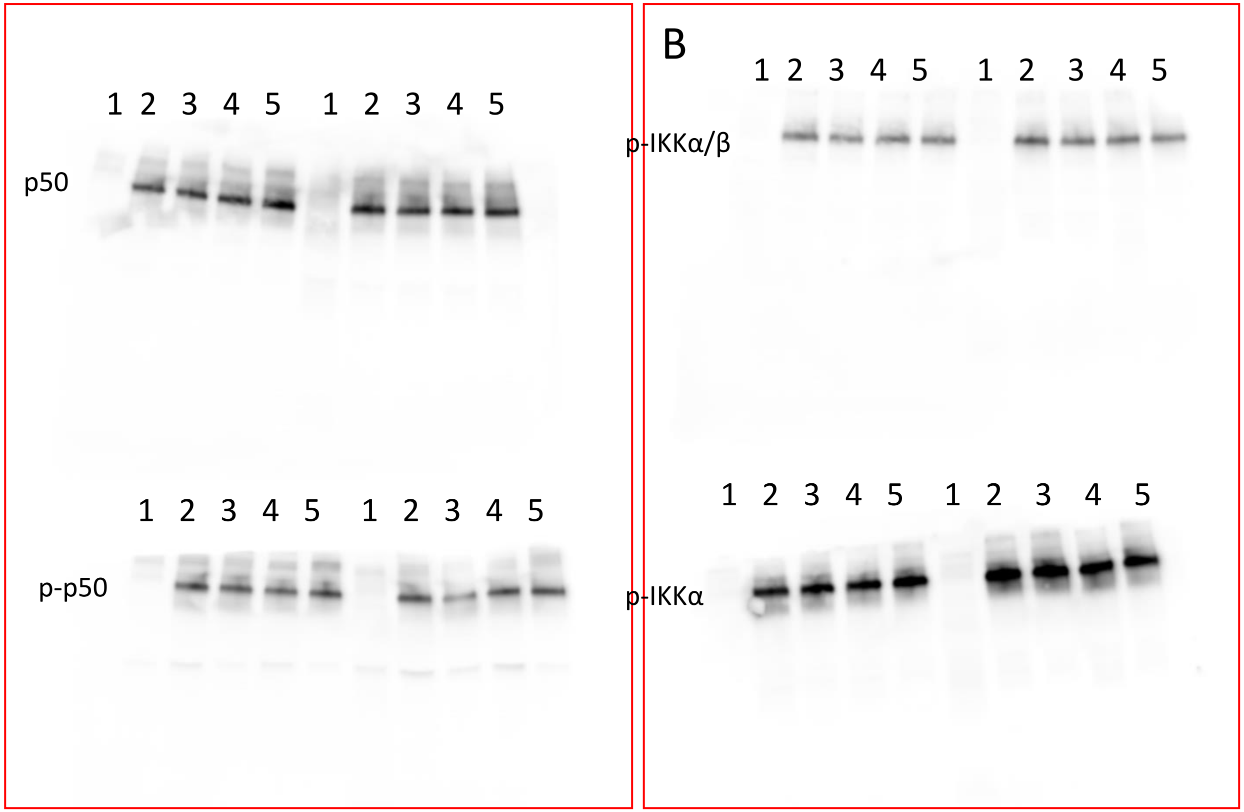

Figure S1b. Effect of gallic acid on NFκB canonical signaling pathway. Human CD14+ monocytes were treated with gallic acid upon *Borrelia burgdorferi* 31 stimulation. Levels of proteins were assessed by Western blot after 12 h post-treatment. 1 - control cells not stimulated with *Borrelia burgdorferi*; 2 - control cells stimulated with *Borrelia burgdorferi* at cells:bacteria 1:5 ratio; 3 - cells stimulated with *Borrelia burgdorferi* at cells:bacteria 1:5 and treated with gallic acid at 25 µg/ml; 4 - cells stimulated with *Borrelia burgdorferi* at cells:bacteria 1:5 and treated with gallic acid at 50 µg/ml; 5 - cells stimulated with *Borrelia burgdorferi* at cells:bacteria 1:5 and treated with gallic acid at 75 µg/ml.

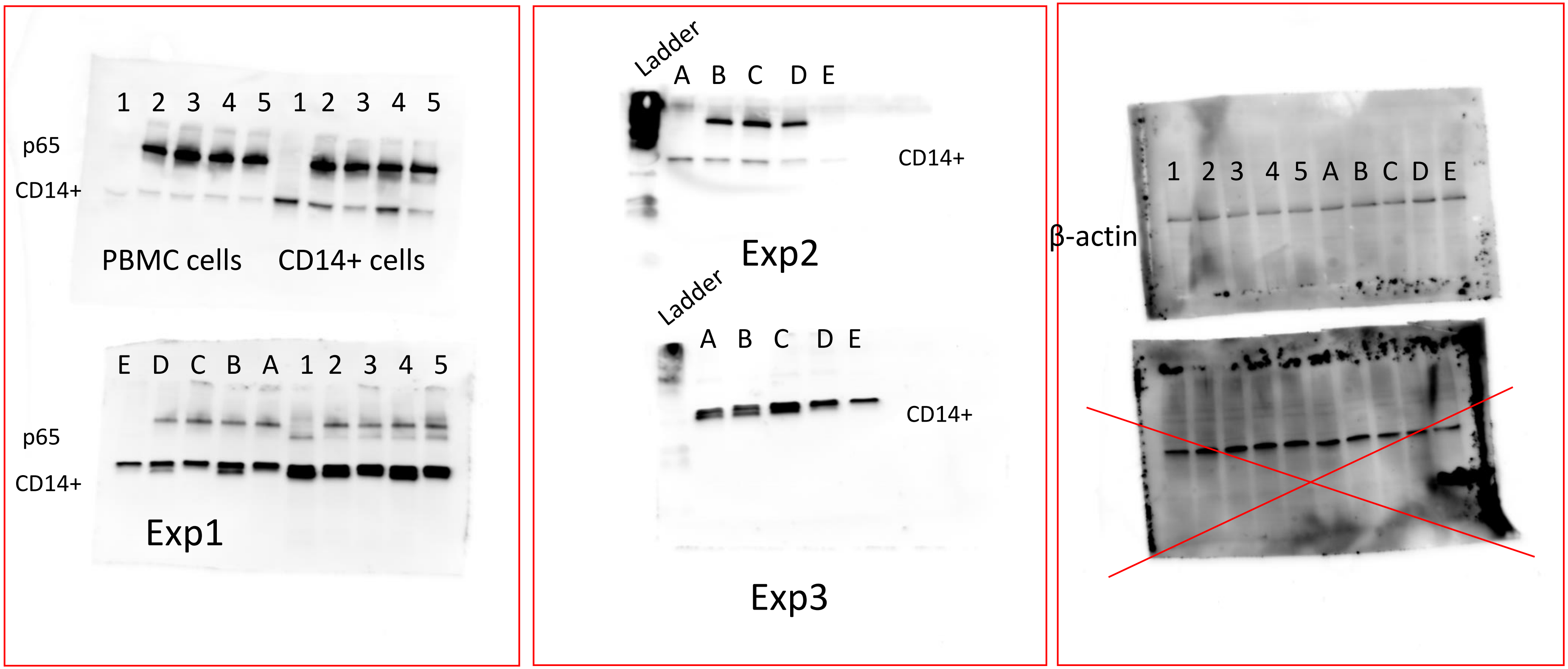

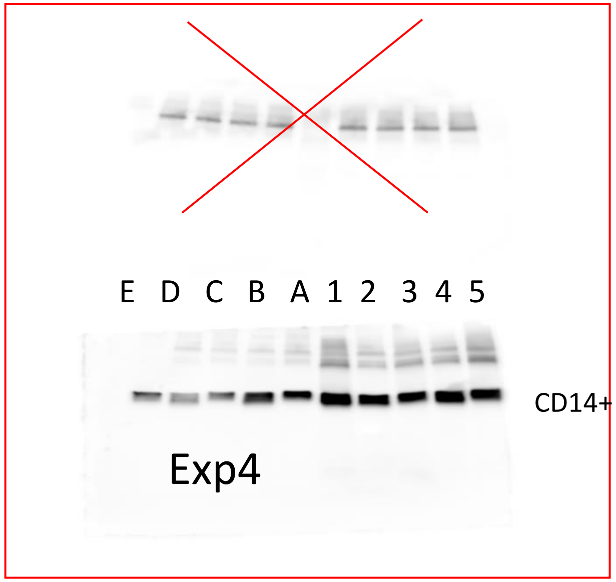

Figure S2. Effect of gallic acid on Toll-like receptor 2. Human CD14^{+} monocytes were treated with gallic acid upon *Borrelia burgdorferi* 31 stimulation. Levels of proteins were assessed Western blot after 12 h post-treatment. 1 - control cells not stimulated with *Borrelia burgdorferi*; 2 - control cells stimulated with *Borrelia burgdorferi* at cells:bacteria 1:5 ratio; 3 - cells stimulated with *Borrelia burgdorferi* at cells:bacteria 1:5 and treated with gallic acid at 50 µg/ml; 4 - cells stimulated with *Borrelia burgdorferi* at cells:bacteria 1:10; 5 - cells stimulated with *Borrelia burgdorferi* at cells:bacteria 1:10 and treated with gallic acid at 50 µg/ml; A - control cells not stimulated with *Borrelia burgdorferi*; B - control cells stimulated with *Borrelia burgdorferi* at cells:bacteria 1:5 ratio; C - cells stimulated with *Borrelia burgdorferi* at cells:bacteria 1:5 and treated with gallic acid at 25 µg/ml; D - cells stimulated with *Borrelia burgdorferi* at cells:bacteria 1:5 and treated with gallic acid at 50 µg/ml; E - cells stimulated with *Borrelia burgdorferi* at cells:bacteria 1:5 and treated with gallic acid at 75 µg/ml.
